# Supplementary material for: Fetal and neonatal outcomes in syphilis infected pregnant women in Reunion Island: An observational retrospective multicentric study
Source: PLoS One. 2024 Nov 25;19(11):e0309828. doi: 10.1371/journal.pone.0309828 (PMC11588228; doi:10.1371/journal.pone.0309828)
Supplement: Attachment — Submitted filename: Response to reviewer 260224.docx [file pone.0309828.s003.docx]

***Mrs Irene Nathalie Fernandez Tolentino
PLOS ONE***

Object : submission of the revised version of the manuscript PONE-S-24-00341

Dear Editorial board of PLOS ONE,

On behalf of all authors of the above-mentioned manuscript, we would like to thank you and the reviewer for your insightful feedback of the article entitled “**Fetal and neonatal outcomes in syphilis infected pregnant women in Reunion Island”**. You will find below the responses to the respective suggestions made by the reviewers. We hope that you will be pleased with this revised, enriched version of the manuscript and we look forward to hearing from you.

Yours faithfully,

*Changes have been made accordingly and four sentences were added p.4, l. 95 ; tracked changes are highlighted in yellow.*

**Reviewers' comments:**

- Please provide additional details regarding participant consent. In the ethics statement in the Methods and online submission information, please ensure that you have specified (1) whether consent was informed and (2) what type you obtained (for instance, written or verbal, and if verbal, how it was documented and witnessed). If your study included minors, state whether you obtained consent from parents or guardians. If the need for consent was waived by the ethics committee, please include this information.

  If you are reporting a retrospective study of medical records or archived samples, please ensure that you have discussed whether all data were fully anonymized before you accessed them and/or whether the IRB or ethics committee waived the requirement for informed consent. If patients provided informed written consent to have data from their medical records used in research, please include this information

  Once you have amended this/these statement(s) in the Methods section of the manuscript, please add the same text to the “Ethics Statement” field of the submission form (via “Edit Submission”).

  For additional information about PLOS ONE ethical requirements for human subjects research, please refer to <http://journals.plos.org/plosone/s/submission-guidelines#loc-human-subjects-research>.

*During consultation, verbal consent was obtained from patients to transcript their history in their medical records. Then, for this retrospective study of medical records, all data were fully anonymized before we accessed them. Authors had not access to information that could identify individual participants during or after data collection.*

*Data were accessed for research purposes on May, 1 2023.*

*Minors were excluded from our study.*

*These sentences were added in the new version of the uploaded manuscript and in the “Ethics Statement” field of the submission form.*

- Please complete all items on the Human Participants Research Checklist that are relevant for your submission, by following this link:  <http://journals.plos.org/plosone/s/file?id=dc11/PLOSOne_Human_Subjects_Research_Checklist.docx> (Contact us at [plosone@plos.org](mailto:plosone@plos.org) if you cannot access the document.) There may be overlap between the checklist items and other queries listed below; please address any duplicated queries both in your response email and on the checklist itself. Upload the completed Human Participants Research Checklist as file type “Other” when you re-submit your manuscript. This document is for internal journal use only and will not be published if your article is accepted. The requested information will help us to assess whether your submission complies with PLOS ONE’s policies and adheres to applicable reporting standards. Note that your manuscript may be rejected if you provide incomplete or inadequate responses to the checklist questions and that changing the ‘Section/Category’ of your article does not affect this requirement.

*All items on the Human Participants Research Checklist that are relevant for submission were completed and this document is uploaded as file type “Other”.*

*Moreover, ethics approval by the Research Ethics Committee of the University Hospital of Bordeaux was uploaded, with English translate.*

- Please remove your figures from within your manuscript file, leaving only the individual TIFF/EPS image files.  These will be automatically included in the reviewer’s PDF.

  We've returned your manuscript to your account. Please resolve these issues and resubmit your manuscript within 21 days. If you need more time, please email the journal office at [plosone@plos.org](mailto:plosone@plos.org). We are happy to grant extensions of up to one month past this due date. If we do not hear from you within 21 days, we will withdraw your manuscript.

  Please log on to PLOS Editorial Manager at <https://www.editorialmanager.com/pone/> to access your manuscript. You will find your manuscript in the 'Submissions Sent Back to Author' link under the New Submissions menu. Be sure to remove your previous manuscript file if you are uploading a new file in response to these requests. After you've made the changes requested above, please be sure to view and approve the revised PDF after rebuilding the PDF to complete the resubmission process.

  We are requesting these changes to comply with the PLOS ONE submission guidelines (<https://journals.plos.org/plosone/s/submission-guidelines>). Please note that we won't send your manuscript for review until you have resolved the above requests.

*The two figures (Figure 1 – Fetal and neonatal mortality and morbidity according to ultrasound follow-up and* *Figure 2 – Fetal and neonatal mortality and morbidity according to treatment) were removed from manuscript file. They can be found in two separate files, one file per figure, named “Figure 1 – US anomalies” and “Figure 2 – Treatment”.*

**Update 26/02/2024**

- We noticed that you have not returned the Human Participants Research Checklist. Please note that the completion of this checklist is required and should it fail to be returned, your submission will be withdrawn.

*All items on the Human Participants Research Checklist that are relevant for submission were completed and this document is uploaded as file type “Other”.*

- Please upload a clean copy of your manuscript that does not contain any track changes. Upload this as your “Manuscript” file.

*Track changes were removed and a clean copy of the manuscript is uploaded as my “Manuscript” file.*
