# Supplementary material for: Fetal and neonatal outcomes in syphilis infected pregnant women in Reunion Island: An observational retrospective multicentric study
Source: PLoS One. 2024 Nov 25;19(11):e0309828. doi: 10.1371/journal.pone.0309828 (PMC11588228; doi:10.1371/journal.pone.0309828)
Supplement: Attachment — Submitted filename: Response to reviewers 050724.docx [file pone.0309828.s004.docx]

***Oriel Jerome Delas Alas Vida***

***Academic Editor ;
PLOS ONE***

Object: submission of the revised version of the manuscript PONE-D-24-05431R1

Dear Editorial board of PLOS ONE,

On behalf of all authors of the above-mentioned manuscript, we would like to thank you and the reviewer for your insightful feedback of the article entitled “**Fetal and neonatal outcomes in syphilis infected pregnant women in Reunion Island”**. You will find below the responses to the respective suggestions made by the reviewers. We hope that you will be pleased with this revised, enriched version of the manuscript and we look forward to hearing from you.

Yours faithfully,

*Changes have been made accordingly and sentences have been changed in lines 145, 147, 150, 163, 164, 188 and 201. You may find a copy of the manuscript with tracked changes highlighted in yellow, named as “Revised Manuscript with Track Changes”.*

You will find, in the revised submission, as attached files:

- The revised manuscript, named as “Manuscript”
- A copy of the manuscript with tracked changes highlighted in yellow, named as “Revised manuscript with track changes”.
- This letter “Response to reviewers 240524”.
- The two figures in separate files, named “fig1.tif” and “fig2.tif”, revised with the reviewer suggestion and accordingly PLOS ONE Guidelines
- The Excel document with anonymized data
- The tables with all statistical analysis

**Reviewers’ question in July, 5^th^ 2024 :**

We note you updated your Data Availability statement to:

"Authors agree to make their data available upon reasonable request by writing to [camille.cramez@chu-reunion.fr](mailto:camille.cramez@chu-reunion.fr). However, for third-party data that authors cannot legally distribute :
1. data set include same information than in our data set, though with patients from other hospitals
2. authors obtained permission to use the data set
3. authors did not receive any special privileges in accessing the data that other researchers would not have
4. if readers need to apply to gain access to the data, they may contact [pi.rouffet@chor.re](mailto:pi.rouffet@chor.re)"

As we stated previously, PLOS’ Data Policy states that authors cannot be the sole named individuals responsible for ensuring data access.

Therefore, please confirm if interested researchers may contact the provided email address "[pi.rouffet@chor.re](mailto:pi.rouffet@chor.re)" to request the data relevant to this study?

If so, please clarify who is the recipient of the email address and what are their role/title and institution.

We will update your Data Availability statement on your behalf using the information you provide.

- *The recipient of the email address is Dr Pierre ROUFFET, who is the pedagogical coordinator and the head of the Medical Information Department (DIM, in French), Centre Hospitalier Ouest Réunion (CHOR). He provided data from the Hospital in west Reunion Island, which has a different computer software from the University Hospitals in North and South Reunion Island. Data from the hospital located in East Reunion is linked with the University Hospital in North Reunion Island.*

**Reviewers’ question in June, 24th 2024:**

Concerning data availability, some restrictions will apply. To justify, see the paragraph added in Methods, as written below.

**Reviewers’ question in June, 6th 2024:**

- ***Data availability statement :***

A new paragraph was added in Methos :

Authors agree to make their data available upon reasonable request by writing to [camille.cramez@chu-reunion.fr](mailto:camille.cramez@chu-reunion.fr)

However, for third-party data that authors cannot legally distribute :

1. data set include same information than in our data set, though with patients from other hospitals
2. authors obtained permission to use the data set
3. authors did not receive any special privileges in accessing the data that other researchers would not have
4. if readers need to apply to gain access to the data, they may contact [pi.rouffet@chor.re](mailto:pi.rouffet@chor.re)

- ***Please include a separate legend for each figure in your manuscript.***

The titles have been retired from the figures and included in another file.

**Reviewers' comments and suggestion in May, 2024:**

1) Reviewer: If you would like to make changes to your financial disclosure, please include your updated statement in your cover letter.

- *Authors have no financial disclosure to declare.*

2) Reviewer: We recommend that you deposit your laboratory protocols in [protocols.io](http://protocols.io/) to enhance the reproducibility of your results.

- *The laboratory protocol has been deposited on protocols.io under and is successfully published, under the DOI : https://dx.doi.org/10.17504/protocols.io.6qpvr83mblmk/v1*

3) Reviewer: PLOS ONE's style requirements, including those for file naming. The PLOS ONE style templates can be found at
   <https://journals.plos.org/plosone/s/file?id=wjVg/PLOSOne_formatting_sample_main_body.pdf> and 
   <https://journals.plos.org/plosone/s/file?id=ba62/PLOSOne_formatting_sample_title_authors_affiliations.pdf>

- *Manuscript has been revised according to PLOS ONE Style Requirements.*

*The first page, with authors affiliation, has been changed, with 2 e-mail addresses left (Phuong Lien TRAN and Camille CRAMEZ). The mention “MD” and “PhD” have been removed. We wanted to mention that Camille CRAMEZ, Marine LAFONT and Phuong Lien TRAN contributed equally to this work.*

4) Reviewer: We note that you have indicated that there are restrictions to data sharing for this study. For studies involving human research participant data or other sensitive data, we encourage authors to share de-identified or anonymized data. However, when data cannot be publicly shared for ethical reasons, we allow authors to make their data sets available upon request. For information on unacceptable data access restrictions, please see <http://journals.plos.org/plosone/s/data-availability#loc-unacceptable-data-access-restrictions>.

Before we proceed with your manuscript, please address the following prompts:

If there are ethical or legal restrictions on sharing a de-identified data set, please explain them in detail (e.g., data contain potentially identifying or sensitive patient information, data are owned by a third-party organization, etc.) and who has imposed them (e.g., a Research Ethics Committee or Institutional Review Board, etc.). Please also provide contact information for a data access committee, ethics committee, or other institutional body to which data requests may be sent.

If there are no restrictions, please upload the minimal anonymized data set necessary to replicate your study findings to a stable, public repository and provide us with the relevant URLs, DOIs, or accession numbers. Please see <http://www.bmj.com/content/340/bmj.c181.long> for guidelines on how to de-identify and prepare clinical data for publication. For a list of recommended repositories, please see <https://journals.plos.org/plosone/s/recommended-repositories>. You also have the option of uploading the data as Supporting Information files, but we would recommend depositing data directly to a data repository if possible.

“The [PLOS Data policy](http://www.plosone.org/static/policies.action#sharing) requires authors to make all data underlying the findings described in their manuscript fully available without restriction, with rare exception (please refer to the Data Availability Statement in the manuscript PDF file). The data should be provided as part of the manuscript or its supporting information, or deposited to a public repository. For example, in addition to summary statistics, the data points behind means, medians and variance measures should be available. If there are restrictions on publicly sharing data—e.g. participant privacy or use of data from a third party—those must be specified. »

- *After discussion between the authors, it did not seem unreasonable to release the data, given that no institution has forbidden it, subject to correct anonymization. Please find attached the Excel document with anonymized data collection and statistical tables.*

5) Reviewer: Please amend either the title on the online submission form (via Edit Submission) or the title in the manuscript so that they are identical

- *It is done, the two titles are identical, sorry for the oversight.*

6) Reviewer: Please review your reference list to ensure that it is complete and correct. If you have cited papers that have been retracted, please include the rationale for doing so in the manuscript text, or remove these references and replace them with relevant current references. Any changes to the reference list should be mentioned in the rebuttal letter that accompanies your revised manuscript. If you need to cite a retracted article, indicate the article’s retracted status in the References list and also include a citation and full reference for the retraction notice.

- *All reference have been reviewed and can be found on the web. They all are recent articles. If you do not find one of them, may I ask you to tell me, so that I could help you?*

7) Reviewer: modifications in more literary English

144: No re-ascension would be better stated as “no rise in VDRL titers”

158: “the number of injections was adapted to the stage of syphilis” could be better stated as if the treatment was appropriate for stage of syphilis.

181-182: for an English audience, “protected their sexual relations” is also a little oddly put, “had protected intercourse”, or simply “used protection consistently”.

197: looks like 16SA needs to be 16 WG (again this is also up at line 146)

Figure 1: I think I understand what the authors mean by with and without congenital syphilis, the problem with this is the probabilistic nature of the infection they note earlier in the manuscript. I would recommend “not proven” or something of the like. Alternatively could expand out to have all 4 scenarios.

- *Changes have been made.*

163: this reads poorly, I would work this sentence into the preceding paragraphs

- *The sentence has been modified: “Follow-up of syphilis during pregnancy was considered optimal if the patient was properly addressed to infectious disease (CEGGID) and Antenatal Diagnosis (DAN) departments, with monthly ultrasound and VDRL, but also if psychosocial care was provided, according to the regional protocol.”*

General terms of vulnerable socio-ecomonic background are hard to interpret given that there appears to be no clear definition given in the paper.

180-181: While I can assume what one would mean by a “vulnerable psycho-social background” by reading the table, it might be good to define or state within the text.

- *A definition for the term “vulnerable socio-economic background” has been added : “A woman was identified with a vulnerable psycho-social background when she met any of the following criteria: domestic violence, incarcerated partner or partner with poly-intoxication, history of sexual assault or multiple abortions, psychological disorders, children in care or out of school, medico-psycho-social hospital file or housing difficulties.”*

8) Reviewer: 88-89: authors report that there is systematic screening which is well conducted, and would like to see some numbers which relate to this assertion. In the US many states have mandated screening three times in pregnancy, and despite laws being in place there have been surges suggesting that screening may not be as good as they/we expect.

*The 2021 La Réunion perinatal survey revealed that almost 99% of pregnant women were screened for syphilis. You may see on table 19 below that between 98 and 99.9% of pregnant women have been screened for syphilis in La Réunion. You may find the percentage for mainland France just at the right of these numbers.*

<https://www.santepubliquefrance.fr/determinants-de-sante/sante-sexuelle/documents/enquetes-etudes/sante-perinatale-a-la-reunion.-resultats-de-l-enquete-nationale-perinatale-2021-dans-les-drom-enp-drom-2021>

9) Reviewer: « Excluding pregnancy with missing neonatal data may bias these results, curious if there is an approximation of how many pregnancies had missing neonatal data and why this would be the case. I am assuming that perhaps this means any follow up data at 28 days of age, which if so, might just be good to just state as such, because missing neonatal data could be weight, or length, or gestational age, etc..”

- *Unfortunately, it is true that it may bias our results.*

*On 124 patients screened by the Department of Medical Information (DIM) and in the CPDPN files, 11 must have been excluded because medical files were totally incomplete. In La Réunion, medical files are informatized and it was not acceptable that the information was not completely filled in.*

*Moreover, 5 patients intended to be included have to be excluded because neonatal data missed such as, indeed, birth weight, birth length, birth cranial perimeter, neonatal exams to determine the CDC scenario. It can be explained by two factors in addition of an uncompleted file by human error. The first one is the lack of experience in case of suspicion of congenital syphilis in small centers, outside the University Hospital. The second one is that when newborns are taken into intensive care, all the biometrics are not measured in delivery room and then theses information lack in informatized files.*

*I hope this sounds clear to you.*

10) Revi ewer: I am interested to see so many individuals with “congenital syphilis unlikely” as noted in table 6. If many of these women were receiving treatment during pregnancy, then per the CDC guideline, the children could not be classified as such as the guideline states “Scenario 4: Congenital Syphilis Unlikely
    Any neonate who has a normal physical examination and a serum quantitative nontreponemal serologic titer equal to or less than fourfold of the maternal titer at delivery and both of the following are true:
    The mother’s treatment was adequate before pregnancy. The mother’s nontreponemal serologic titer remained low and stable (i.e., serofast) before and during pregnancy and at delivery (e.g., VDRL ≤1:2 or RPR ≤1:4). I am totally open to the possibility that I am misinterpreting this but in general treatment during pregnancy precludes being classified as unlikely. I do not feel that this appreciably changes the results from the paper, but I do think it is worth mentioning. Furthermore, if the

- *I understand what you mean, but as the CDC Scenario 4 is defined as: "…. a newborn as described in scenario 3, but whose mother was properly treated before 16WG, with stable, low VDRL follow-up” (l. 146). So maybe I am misinterpreting but an appropriate treatment during pregnancy does not prevent from being classified as CDC Scenario 4. My hypothesis is that the word “unlikely” is maybe poorly translated from French to English. I would have preferred “low probability scenario” but the word “unlikely” had been choose by the CDC guidelines. I totally agree with you that the risk of congenital syphilis of a child classified in CDC Scenario 4 is not totally equal to zero. However, the probability is weak, upon to me.*

*Thank you for mentioning this issue because it troubled me also.*

11) Reviewer: « While revising your submission, please upload your figure files to the Preflight Analysis and Conversion Engine (PACE) digital diagnostic tool, <https://pacev2.apexcovantage.com/>. PACE helps ensure that figures meet PLOS requirements. To use PACE, you must first register as a user. Registration is free. Then, login and navigate to the UPLOAD tab, where you will find detailed instructions on how to use the tool. If you encounter any issues or have any questions when using PACE, please email PLOS at [figures@plos.org](mailto:figures@plos.org). Please note that Supporting Information files do not need this step. »

*The two figures have been downloaded and converted on PACE, guaranteeing figures meet PLOS requirements. The are uploaded in two different files under the name “fig1.tif” and ‘fig2.tif”.*
